# Phagocytic Function and Flow Cytometric Phenotype of Asian Elephant Monocytes

**DOI:** 10.3390/ani14162297

**Published:** 2024-08-07

**Authors:** Jennifer L. Johns, Trinity R. Baumgartner, Carlos R. Sanchez, Brian P. Dolan

**Affiliations:** 1 Department of Biomedical Sciences, Carlson College of Veterinary Medicine, Oregon State University, Corvallis, OR 97331, USA; baumgart@oregonstate.edu (T.R.B.); brian.dolan@oregonstate.edu (B.P.D.); 2 Oregon Zoo, Portland, OR 97221, USA; carlos.sanchez@oregonzoo.org

**Keywords:** Asian elephant, monocyte, flow cytometry, phagocytosis, IBA1

## Abstract

**Simple Summary:**

All elephant species are now endangered or critically endangered. It is extremely important for the veterinary care of elephants to be effective at diagnosing diseases and monitoring treatment. Routine blood cell testing is a standard method for monitoring elephant health and detecting possible infections. Elephants naturally have different ratios of white blood cells (WBCs; immune cells) in their blood compared to other animals, with unusually high percentages of one cell type (monocytes). Because elephant WBCs also look different from those of most other animals, a person must identify the cells under the microscope instead of using machine analysis. These elephant-specific WBC features may be important in fighting diseases, but very little is known about elephant monocytes. The aims of this study were to establish methods for isolating elephant monocytes, studying their function, and accurately identifying them using flow cytometry, a more precise technique than microscopy. Blood from Asian elephants was used. Methods were created and tested. An approach to identifying elephant monocytes using flow cytometry was established. These results will lead to better studies of the elephant immune system and can help to more accurately count WBCs in elephant samples.

**Abstract:**

Optimal veterinary care of managed elephant populations is vital due to the continued decline of wild populations. Appropriate health monitoring and accurate disease diagnosis include hematologic evaluation. Elephant hematology is distinctive in that elephants have high percentages of monocytes in health. Elephant monocytes also have unusual morphology, a feature shared with manatees and rock hyraxes. Manual white blood cell counting is used for elephant hematology, as analyzers are generally inaccurate. The aims of this study were to evaluate basic cell isolation and functional testing protocols for use in elephant monocyte research, and to test several available antibodies via flow cytometry for use in elephant monocyte identification. Peripheral blood samples from five Asian elephants (*Elephas maximus*) were used. Methods for monocyte isolation and evaluation of phagocytic function were established. Putative lymphocyte and monocyte populations were identified using a scatter on flow cytometry. Antibodies against CD11b, CD11c, CD14, and ionized calcium-binding adapter molecule 1 (IBA1) were tested, with IBA1 showing the highest apparent diagnostic utility in labeling monocytes. Combined flow cytometric scatter and IBA1 positivity appear to identify Asian elephant monocytes. These data provide a methodologic basis for further investigation into elephant monocyte function and immune response to infection.

## 1. Introduction

Of the three elephant species extant on our planet, all are now listed as endangered or critically endangered [1]. Habitat loss, poaching, and other human interference greatly threaten elephant survival, but naturally occurring infectious and inflammatory diseases also impact populations. Elephant endotheliotropic herpesvirus (EEHV) can cause acute fatal hemorrhagic disease (EEHV-HD) in young Asian elephants (*Elephas maximus*) and African elephants (*Loxodonta africana* and *L. cyclotis*) [2,3]. *Mycobacterium tuberculosis* infection in captive/managed Asian elephants can result in chronic wasting disease, is challenging to eliminate from herds, and poses a zoonotic threat to humans that can force euthanasia of infected but healthy elephants [4]. Additionally, captive elephants in range countries are often in contact with free-living elephants, raising the potential of tuberculosis spread to wild animals.

Accurate diagnosis, treatment, and management of diseases in elephant populations is critical for their continued survival. Routine hematologic monitoring of captive elephants is a standard of care, and early hematologic evaluation of clinically ill elephants can determine the presence/severity of systemic inflammation and the treatment approach [5,6]. Elephants have unusual blood cell morphology, specifically in monocytes, and most hematology analyzers cannot accurately identify elephant leukocyte subtypes [7,8]. Some published reference intervals for elephant hematology are inaccurate for this reason [9]. Also, elephant “differentials” (percentage of each leukocyte type in circulation) are very different from other mammals. Monocytes comprise the majority of all circulating elephant leukocytes, in comparison to the typical 2–4% of leukocytes seen in other mammals [8,10,11]. Although a few closely related species, such as manatees and rock hyraxes, have similarly atypical monocyte morphology, none have similarly high monocyte percentages of peripheral blood leukocytes [12,13,14,15].

The importance of this highly unusual monocyte predominance in the elephant immune response to infection is unknown. It may be relevant particularly in EEHV infection, as monocyte levels correlate closely with onset of severe disease and with recovery [16,17]. Minimal characterization of elephant monocytes (apart from cytochemical staining) has been published, and species-specific reagents are not commercially available. Additionally, manual differential counts are required for elephant hematology, but high analytical variation inherent in such counts can be problematic [18]. Flow cytometric profiling has the potential to increase accuracy and to identify phenotypic subsets. The elephant leukogram is reported to often remain unchanged until late in some disease processes [6], but more accurate cell identification could provide earlier detection of leukocyte shifts. One publication describes elephant T-lymphocyte phenotyping via flow cytometry [19], and a few publications describe immunohistochemical labeling of elephant lymphocytes or macrophages [16,20], but no references to monocyte characterization via flow cytometry are available.

The objectives of this study were (1) to establish methods for isolation of Asian elephant monocytes and assessment of phagocytic ability, and (2) to test commercially available monoclonal antibodies in phenotypic characterization of Asian elephant monocytes. This work will create a methodologic basis for further functional studies.

## 2. Materials and Methods

### 2.1. Animal Population, Sample Information, and Institutional Approval

Research was conducted in accordance with institutional Animal Care and Use regulations and the U.S. Animal Welfare Act. Blood collection and use was approved by the Institutional Review Board at Oregon State University (exemption issued) and the Oregon Zoo Research Review Committee. Whole blood samples were obtained from Asian elephants (*E. maximus*) at the Oregon Zoo. Samples were obtained from the five adult Asian elephants currently at the zoo during routine phlebotomy for regular hormonal monitoring. The elephants are three males (indicated as M1, M2, and M3) and two females (indicated as F1 and F2; F2 is a Borneo elephant subspecies, *E. maximus borneensis*). Equine whole blood was obtained from the Oregon Veterinary Diagnostic Laboratory. Samples were initially collected for clinical diagnostic use in the OSU Veterinary Teaching Hospital (VTH). Sample use for this study was permitted via OSU VTH client consent forms.

### 2.2. Isolation of Peripheral Blood Mononuclear Cells (PBMCs)

Blood smears were prepared from peripheral blood anticoagulated with EDTA (ethylenediaminetetraacetic acid; BD Vacutainer EDTA tubes, BD, Franklin Lakes, NJ, USA) and stained with modified Wright stain (Camco quick stain, Cambridge Diagnostic Products Inc., Fort Lauderdale, FL, USA). Remaining blood was overlaid on specific gravity 1.077 Ficoll-Paque PLUS (Cytiva, Marlborough, MA, USA) in a ratio of 1-part EDTA-anticoagulated whole blood to 1.25-parts Ficoll-Paque. Blood volume used varied from 10 to 13 mL. Tubes were centrifuged at 400× *g* for 40 min at room temperature (22 °C) with zero brake. Plasma was removed and banked, and the PBMC layer was harvested. Sterile Dulbecco’s PBS (DPBS, Thermo Fisher Scientific, Waltham, MA, USA) was added, and tubes were centrifuged at 500× *g* for 8 min at room temperature. Supernatant was removed and pellets were resuspended in sterile DPBS and centrifuged at 150× *g* for 8 min to remove platelets. Supernatant was removed and pellets were resuspended in sterile cell culture media. Media used for all cell culture was low-glucose DMEM (Corning Inc., Corning, NY, USA) with 10% fetal bovine serum (Avantor, Radnor Township, PA, USA), 1% *v*:*v* 200 mM l-glutamine (Corning Inc.), and 1% *v*:*v* penicillin/streptomycin (Corning Inc.). PBMC cell concentration was assessed via hemocytometer count. To confirm PBMC purity, cytocentrifuge smears were made (Shandon Cytospin 4, Thermo Fisher Scientific) and stained with modified Wright stain. In total, 200-cell differential counts were performed on smears. PBMCs from elephant and equine blood were used immediately in flow cytometry assays. For other assays, elephant PBMCs were plated as indicated below, using adherent tissue culture-treated plates (Corning Inc.), and were incubated at 37 °C in 5% CO_2_.

### 2.3. Monocyte Isolation and Dissociation

PBMCs were plated in 24-well tissue culture-treated plates at 0.5 × 10^5^ cells per well and incubated for 24 h prior to dissociation. Media and non-adherent cells were removed via two gentle washes with sterile DPBS, and cytocentrifuge smears were prepared and stained with modified Wright stain for differential counting. Four dissociation protocols (Accutase, lidocaine buffer, EDTA, and dilute trypsin-EDTA) were attempted on remaining adherent monocytes (Supplementary Information—Methods). After incubation, all plates were examined for remaining adherent cells. If many adherent cells remained, plates were placed on an orbital shaker at speed 4 for 10 min, and then were re-examined. Cytocentrifuged smears were prepared from suspensions of dissociated cells and stained with modified Wright stain for differential counting.

### 2.4. Particle Phagocytosis for Spectrophotometry

PBMCs were plated in 96-well plates at 0.1 × 10^5^ cells per well. Following 24 h of incubation, non-adherent cells were removed with two gentle DPBS washes. Zymosan phagocytosis particles (pHrodo Red BioParticles, Invitrogen, Thermo Fisher Scientific) were resuspended to 0.5 mg/mL in sterile DPBS and sonicated for 20 min. Particle suspensions were added sterile DPBS with 10% *v*:*v* elephant plasma and 1% *v*:*v* 200 mg/mL dextrose (“phagocytosis buffer”). Initially, multiple concentrations of particles (50 μL, 25 μL, and 12.5 μL of particle suspension per 500 μL of phagocytosis buffer) were tested (Supplementary Figure S1).

Experiments were performed using 30 μL of particles mixed in 500 μL of phagocytosis buffer (5.7% *v*:*v* particle suspension). A 96-well plate was plated with PBMCs from each of three elephants (F1, F2, M1; 10 wells per elephant sample) and incubated 24 h. Media was removed from monocytes in plates, wells were washed once with sterile DPBS, and particles in phagocytosis buffer were added at 150 μL per well (96-well plate). Negative control samples were monocytes only and particles only. Plates were incubated at 37 °C for 1 h in room air prior to the start of acquisition. Samples were analyzed on a GloMax spectrophotometer (Promega Corp., Madison, WI, USA) at appropriate excitation and emission maxima (627 nm; 580–640 nm). Samples were analyzed every 15 min through 120 min.

### 2.5. Fluorescence Microscopy

PBMCs were plated in sterile gasket wells on coverslips at 20,000 cells/well. After 24 h incubation, wells were washed twice with DPBS, and 40 μL of pHrodo Red zymosan particles in phagocytosis buffer were plated using the 5.7% *v*:*v* suspension described above. Coverslips were incubated as in the above section at 37 °C for 1 h in room air, then gaskets were removed, and coverslips washed three times with sterile DPBS. Nuclei were counterstained with 300 nM DAPI (Thermo Fisher Scientific), and coverslips were mounted with ProLong Gold Antifade mountant (Thermo Fisher Scientific). Slides were imaged on a DMLB microscope (Leica Camera AG, Wetzlar, Germany) with a Leica K3M microscope camera system. Images were acquired via Leica Application Suite X and compiled via GIMP 2.10.36.

### 2.6. Flow Cytometry

Flow cytometry of total Asian elephant and equine white blood cells (WBCs) was performed following lysis of RBCs using ‘ACK buffer’ (0.15 M ammonium chloride, 10 mM potassium bicarbonate, 0.1 mM disodium EDTA). ACK buffer was added to whole blood samples, and then sample were incubated for 5 min in a 37 °C water bath. Samples were then centrifuged at 700× *g* for 5 min and the supernatant was removed. WBCs were washed once with DPBS. Asian elephant PBMCs were isolated via Ficoll procedure as described in Section 2.2. WBCs and PBMCs were counted, as described above, using a hemocytometer.

Cells were resuspended at 250,000 cells/aliquot in flow buffer (sterile DPBS (Ca/Mg-free) with 1% *w*:*v* BSA and 5 mM disodium EDTA) at 100 μL/aliquot in preparation for antibody labeling. The antibodies used (Table 1) were initially optimized using equine WBCs and PBMCs when possible. For antibodies that did not label equine WBCs, a range of concentrations was used when initially tested on elephant WBCs and PBMCs. Experiments were then performed using elephant PBMCs. Samples were incubated with primary antibodies for 30 min at room temperature, and then were washed with flow buffer. Secondary antibodies were added as indicated, and then samples were incubated and washed a second time. Viability dye was then added. In experiments incorporating zymosan particles, pHrodo Green BioParticles (Invitrogen, Thermo Fisher Scientific) in phagocytosis buffer, as described above, were added following the second wash step and samples were incubated at 37 °C for 1 h in room air. Samples were acquired the same day on a FACS Canto analyzer (BD Biosciences, Franklin Lakes, NJ, USA). Data were analyzed using FlowJo v. 10.10 (BD Biosciences). Isotype controls and FMO (fluorescence minus one) samples were used to gate positive and negative fluorescence and set compensation for panels; controls for unconjugated antibodies included cells labeled with secondary antibodies only (Supplementary Figures S2–S4).

### 2.7. Statistical Analysis

Data collation and statistical evaluation were performed using Microsoft Excel (Microsoft 365, Microsoft Corp., Redmond, WA, USA) and GraphPad Prism 10.1 (GraphPad Software, La Jolla, CA, USA). Linear regression analysis was used to compare slopes in spectrophotometric particle phagocytosis assay. Monocyte and lymphocyte percentages obtained via manual microscopic counting and via flow cytometric scatter gating were compared between animals using two-way ANOVA (analysis of variance) for each method. Monocyte and lymphocyte percentages were compared between the two methods using an unpaired *t*-test. Positive and negative events for phenotypic markers listed in Table 1 were compared between monocytes and lymphocytes using a paired *t*-test.

## 3. Results

### 3.1. PBMC Purification via Density Centrifugation

**Figure 1:**
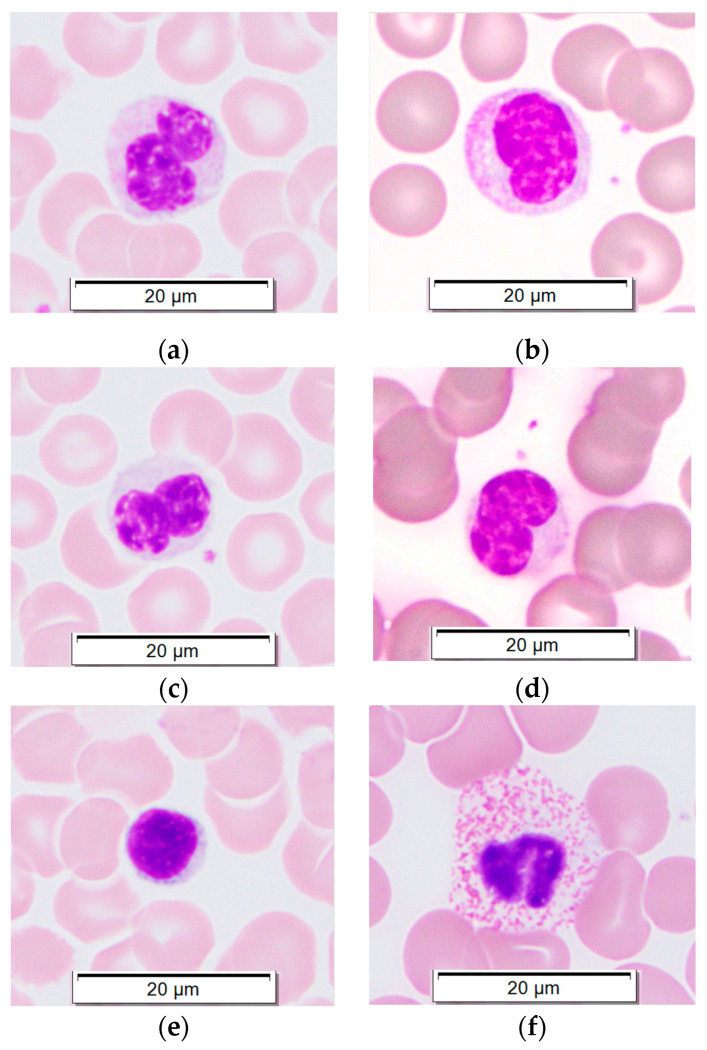

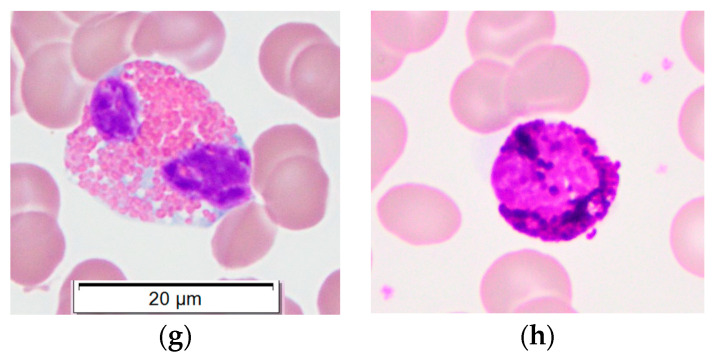
Leukocytes in peripheral blood smears from Asian elephants. Wright-Giemsa stain, 100× magnification. (**a**–**d**) Monocytes; (**e**) Lymphocyte; (**f**) Heterophil (neutrophil); (**g**) Eosinophil; (**h**) Basophil.

**Figure 2:**
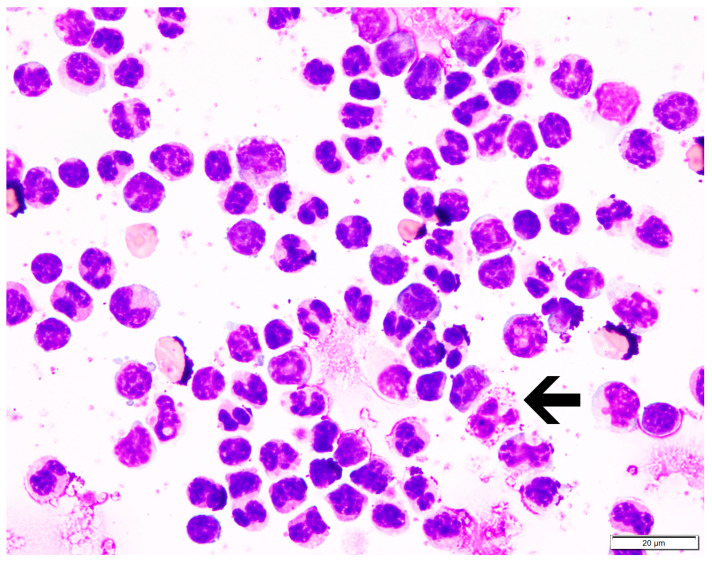
Representative cytocentrifuge smear confirming high PBMC purity following density gradient centrifugation. Modified Wright stain, 100× magnification. Arrow = heterophil.

PBMCs were isolated from five elephants at different points. PBMC purity was 98–99% in all elephant blood samples processed. Monocyte morphology in peripheral blood smears (Figure 1a–d) resembled that of monocytes in post-centrifugation cytocentrifuge smears (Figure 2). Monocytes comprised roughly 70% of all PBMCs, while lymphocytes (Figure 1e) comprised roughly 30%, similar to the ratio of mononuclear cells in peripheral blood. Granulocytes (Figure 1f–h; Figure 2 (arrow)) were rarely present in PBMC preparations.

### 3.2. Monocyte Isolation and Dissociation

Differential counts on cytocentrifuged smears of non-adherent cell suspensions averaged 76% lymphocytes and 24% monocytes, with rare granulocytes seen. Of the four dissociation protocols tested, only dilute trypsin quickly and repeatedly dissociated most adherent cells. The other three dissociation protocols failed to remove many adherent cells despite extended incubation times and use of a plate shaker. Cytocentrifuged smears of dissociated adherent cells using the dilute trypsin protocol contained 100% monocytes/macrophages.

### 3.3. Phagocytosis of Fluorescent Zymosan Particles, Measured Via

#### 3.3.1. Spectrophotometry

**Figure 3:**
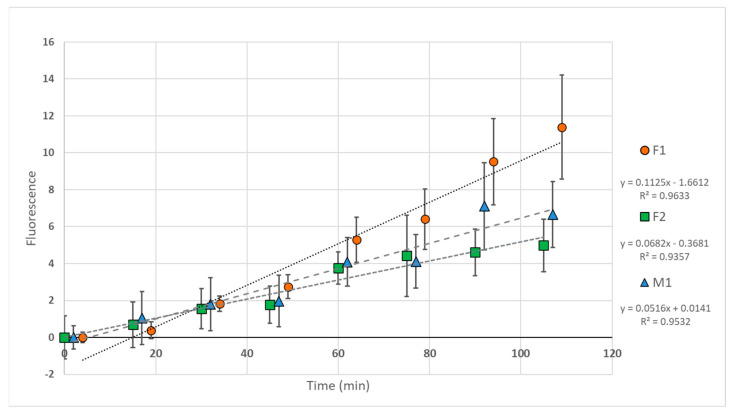
Fluorescence spectrophotometry of elephant monocytes exposed to pHrodo-labeled zymosan particles. Values are normalized to “particle only” negative control wells. Monocytes from three Asian elephants (female 1 (F1), female 2 (F2), and male 1 (M1) were tested. Data points represent mean values; bars represent standard deviation.

Monocytes were isolated from three elephants (F1, F2, M1) and exposed to fluorescent particles. Fluorescence intensity increased over time in all samples (Figure 3), interpreted as particle phagocytosis with subsequent acidification of phagosomes (pHrodo dye fluorescence increases substantially with decreasing pH [21]). Linear regression analysis was used to compare regression line slopes, and all slopes differed significantly: F1 > F2, *p* < 0.0001; F1 > M1, *p* < 0.0001; M1 > F2, *p* = 0.0161.

#### 3.3.2. Fluorescence Microscopy

**Figure 4:**
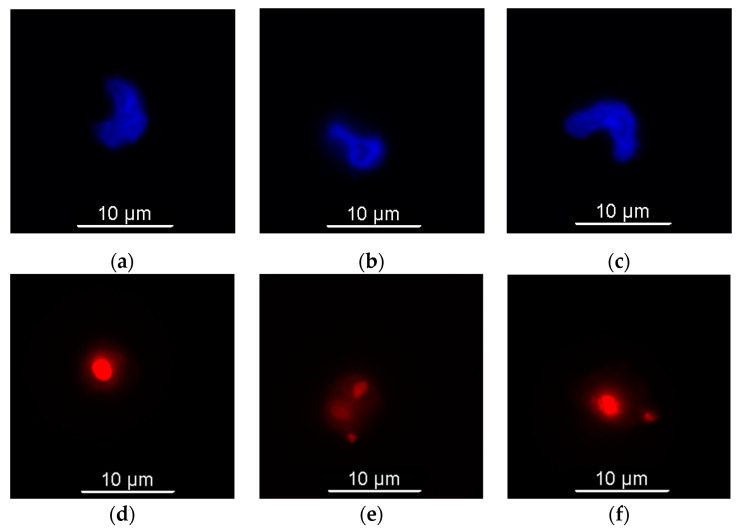

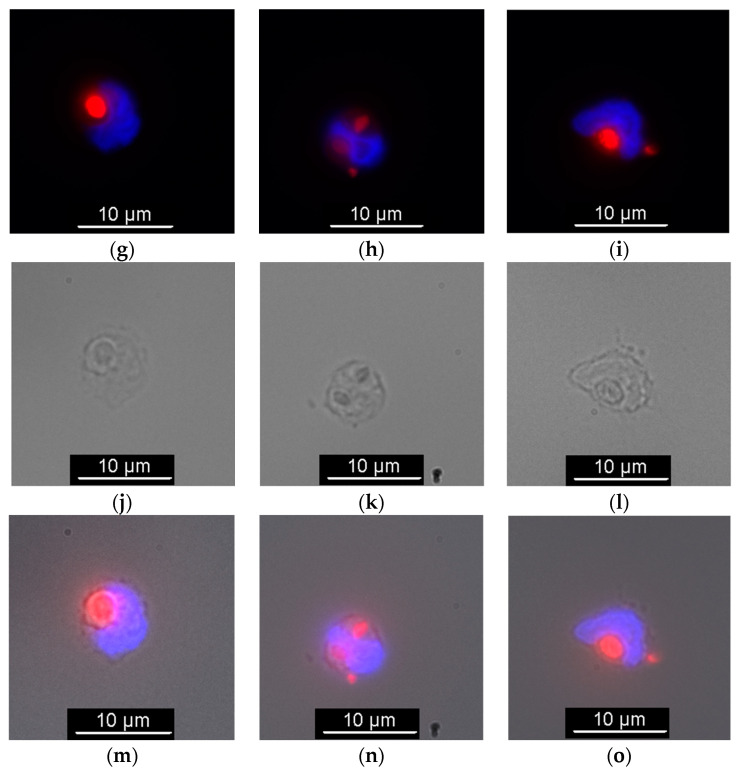
Fluorescence microscopy images of elephant monocytes incubated with zymosan-coated fluorescent particles. (**a**–**c**) DAPI nuclear stain (UV excitation); (**d**–**f**) particle fluorescence (560 nm excitation); (**g**–**i**) merged images of above two rows; (**j**–**l**) contrast images; (**m**–**o**) merged images of all above images.

Monocytes were isolated via plate adhesion using PBMCs isolated from three elephants (F1, F2, M1). In total, 80–90% of all monocytes present in smears from all three elephants contained phagocytosed fluorescent zymosan-coated particles (Figure 4).

### 3.4. Flow Cytometry

#### 3.4.1. Initial Gating

**Figure 5:**
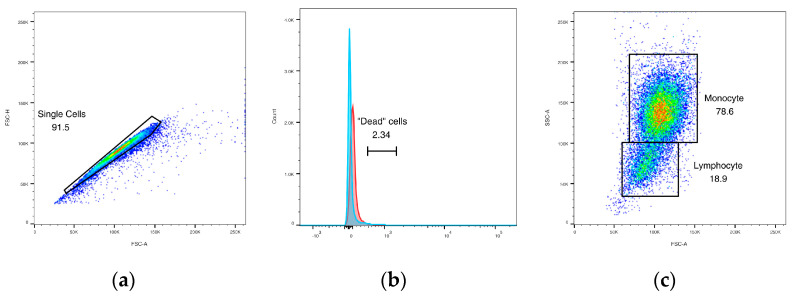
Flow cytometric characterization of Asian elephant peripheral blood mononuclear cells (PBMCs). (**a**) Singlet events gated by forward scatter area (*x*-axis) versus forward scatter height (*y*-axis); (**b**) gating for viability (blue peak = representative sample with viability dye, red peak = sample without viability dye); (**c**) PBMC subsets defined via forward scatter area (*x*-axis) and side scatter area (*y*-axis).

Singlet events were gated using forward scatter area versus height plots (Figure 5a), and then non-viable cells were excluded. Percentage of non-viable cells in all samples was equivalent to that in negative-control samples without viability dye added (Figure 5b), indicating that substantial cell death did not occur during sample handling and processing. Forward scatter area was then plotted against side scatter area. In PBMC samples, two distinct populations were identified as putative lymphocytes vs. monocytes (Figure 5c). Percentages of PBMCs in each population were compared to those obtained via PBMC smear manual differential counts (Table 2). Percentages of each cell type differed significantly between the two methods for both monocytes (*p* < 0.0001, mean difference between means = 9.10) and lymphocytes (*p* = 0.0009, mean difference between means = −4.43). Differences in cell percentages between the three animals were not significant.

#### 3.4.2. Phagocytic Activity

**Figure 6:**
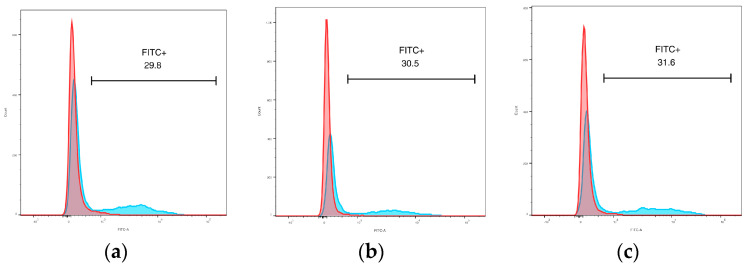
Phagocytosis of zymosan-coated fluorescent particles in lymphocyte and monocyte populations gated via scatter. (**a**) Elephant F1; (**b**) Elephant F2; (**c**) Elephant M1. Red peak = lymphocytes; blue peak = monocytes; “FITC+” = percentage of positive events for particle fluorescence in monocyte population.

When PBMCs were incubated with fluorescent zymosan particles, most cells with positive fluorescence (indicating particle internalization) were found in the monocyte population (Figure 6a–c). The percentage of positive events was compared between monocyte and lymphocyte populations and differed significantly (*p* = 0.0025, mean difference 27.12%, mean ratio of values 9.88).

#### 3.4.3. Surface Phenotype

**Figure 7:**
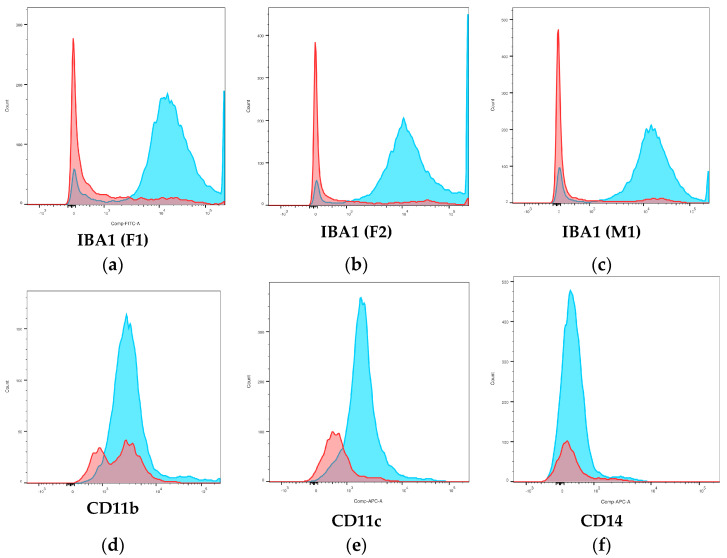
Positive events for surface phenotype markers in lymphocyte and monocyte populations gated via scatter. (**a**) IBA1 positivity, elephant F1; (**b**) IBA1 positivity, elephant F2; (**c**) IBA1 positivity, elephant M1; (**d**) representative histogram for CD11b; (**e**) representative histogram for CD11c; (**f**) representative histogram for CD14. Red peak = lymphocytes; blue peak = monocytes.

The mean percentage of ionized calcium-binding adapter molecule 1-positive (IBA1+) events was 86.8% in monocyte populations and 21.4% in lymphocyte populations gated via scatter of PBMCs, with a significant difference between cell populations (*p* = 0.0088) (Figure 7a–c; Table 3). CD11b+ events were mildly increased in both lymphocyte, and monocyte populations compared to controls and populations did not differ significantly (*p* = 0.1789) (Figure 7d; Table 3). The mean percentage of CD11c+ events was significantly higher in monocytes than lymphocytes (*p* = 0.0499), but peaks overlapped and no distinctive separation between populations was seen (Figure 7e; Table 3). CD14+ events were mildly increased in both lymphocyte and monocyte populations (Figure 7f; Table 3), but populations did not differ significantly (*p* = 0.1119), and staining was variable between experiments. CD115+ events were only slightly increased compared to controls (Supplementary Figure S5; Table 3); populations did not differ significantly (*p* = 0.0982).

## 4. Discussion

This study provides a description of the methodology for isolation and characterization of Asian elephant monocytes. Basic monocyte isolation via plate adhesion and dissociation protocols were established. Phagocytic ability of Asian elephant monocytes was established via fluorescence microscopy, spectrophotometric measurement of fluorescence emission, and flow cytometry. The differentiation of presumed monocyte and lymphocyte populations via flow cytometric scatter was performed. Phagocytic ability and positivity for IBA1 and CD11c were higher in cells identified as monocytes via scatter. Other antibodies against monocyte surface receptors (CD11b, CD14, and CD115) appeared less sensitive and/or specific for monocyte labeling.

IBA1 is a highly conserved marker of monocytes, macrophages, and dendritic cells in multiple species [22,23]. In a prior publication, researchers successfully used a monoclonal anti-IBA1 antibody in immunohistochemistry and immunofluorescence of PBMCs and tissues from EEHV-infected Asian elephants [16]. The antibody clone was not specified, but based on a search of the manufacturer’s website, it appears that it was a different clone from that used in this study. NCBI BLAST was used in this study to identify the degree of protein sequence similarity when selecting antibodies for use. The relatively high degree of similarity in IBA1 (90.6% between *L. africana* and *Homo sapiens*) supports the apparent cross-reactivity of the anti-IBA1 antibody used here in Asian elephant monocytes. Degrees of protein similarity were lower for other antibodies targeting monocytes (CD11b, CD14, CD11c) used in this study, correlating with their apparent lower level of cross-reactivity with Asian elephant monocytes. However, it is possible that other clones may be more sensitive for detection of elephant monocytes and/or more focused flow cytometric testing is needed. CD11c positivity was significantly higher in monocytes than lymphocytes, but the overlap in positivity between populations precluded the use of CD11c alone to differentiate the two populations, and sensitivity for monocytes was apparently moderate. CD11c may prove useful in future phenotypic characterization of elephant monocyte subsets or functional activation.

Specificity of these antibodies for Asian elephant monocytes is a central question. The significant association of IBA1+ and CD11c+ events with cells defined as monocytes by scatter and demonstrating phagocytic ability supports a degree of specific labeling. However, non-specific binding of other WBC subsets remains a concern. Species-specific positive or negative control cell lines (e.g., transfected or knockout cell lines; known positive/negative cells) or competition controls are not available for Asian elephants. Establishment of antibody specificity may rely upon defining all peripheral WBC subsets using antibody panels. Antibodies against T-lymphocyte antigens CD3, CD4, and CD8 have been used in flow cytometry of Asian elephant peripheral blood [19], and CD20 has been used in immunohistochemistry of Asian elephant tissues [20]. Inclusion of these and other lymphocyte-directed antibodies in a larger study would likely help to define the exact specificity of IBA1 and CD11c for Asian elephant monocytes. Additionally, inclusion of more animals and a larger data set could strengthen any associations.

The endangered or critically endangered status of all elephant species places heavy importance on effective conservation efforts and veterinary care. EEHV-HD is the most common cause of mortality in juvenile Asian elephants in captivity [3], and early diagnosis of infection is necessary to initiate appropriate treatment before cellular and organ injury becomes fatal. Peripheral monocytopenia is a documented early marker of hemorrhagic disease and may precede the onset of clinical signs [17]. Accurate quantification of elephant monocytes and other peripheral WBCs is therefore critical for disease surveillance. In the future, establishment and monitoring of leukocyte subsets via flow cytometric phenotyping may allow for more sensitive detection of early EEHV-induced clinical disease. Monocytes are likely involved in the pathophysiology of EEHV-HD as (1) monocytes are likely either infected by EEHV and/or contain phagocytosed viral particles, (2) tissue factor expressed by monocytes is a procoagulant, and (3) macrophages produce pro-inflammatory cytokines (e.g., tumor necrosis factor-alpha and interleukin-6) that promote inflammation and further activation of coagulation [16,24,25]. An improved understanding of elephant monocyte function will support focused research on the mechanisms of disease and appropriate treatment.

Infection with *M. tuberculosis* is another threat to elephant survival. Elephants can develop chronic wasting disease, but clinical signs often do not occur until infection is advanced [26]. Disease surveillance includes trunk washes for culture and/or PCR and serologic monitoring. Treatment has been performed effectively but may be hindered by practical limitations and/or development of drug resistance [27,28]. Zoonotic risk and infection of wild populations are primary concerns. Bidirectional transmission of tuberculosis between captive elephants and humans in close proximity has been demonstrated [29,30], and tuberculosis has been diagnosed in wild Asian and African elephants [31,32]. Given the critical role of macrophages in control of initial infection with *M. tuberculosis* [33], research on elephant monocyte function and macrophage responses to infection may advance diagnostic and treatment strategies for infected elephants.

There were a few practical limitations to this study. It was not possible to compare human protein sequences with those of *E. maximus* via BLAST analysis, but the high degree of genetic similarity between *L. africana* and *E. maximus* supports the use of data from *L. africana* in this approach [34]. The low number of elephants available for sampling precluded generation of reference intervals and most statistical evaluation. Additionally, there were a few potential sources of variability at the animal level. Of the two female elephants, one (F2) is a Borneo elephant, a subspecies shown to be genetically distinct from other Asian elephants and with unknown differences in immune function [35]. The other female elephant (F1) was found to be pregnant, and in retrospect, most samples analyzed from this elephant were obtained during early pregnancy. Effects of elephant pregnancy on immune parameters are generally unknown. Based on studies in pregnant women, monocyte numbers increase in circulation and demonstrate increased activation (e.g., phagocytic activity and generation of reactive oxygen species) [36,37]. It is interesting that the phagocytic rate of macrophages from the pregnant female elephant in this study was higher than that of the other two elephants used for comparison (F2 and M1). This finding may suggest that monocyte/macrophage activation increases during pregnancy in elephants as well, but a larger study is needed to investigate this possibility. Pregnancy can alter monocyte phenotype (e.g., increased CD14 and CD11b expression) as part of pregnancy-associated activation in women [37]. Pregnancy-related phenotypic shifts may also occur in elephant leukocytes; the low sample size of this study precluded evaluation of this question.

Future research in this field can address several gaps in knowledge. Additional methodology should be established to accurately quantify differences in phagocytic function and intracellular killing ability in elephant macrophages. More complete methods for flow cytometric characterization of elephant leukocytes are needed with evaluation of other potentially useful antibodies. To this end, a genomic approach may be helpful to determine the most appropriate targets and species similarities to better inform antibody selection. Following establishment of methods, a primary objective can be the assessment of monocyte and macrophage phenotypic and functional shifts during EEHV infection and tuberculosis in elephants, with the goal of improving veterinary care and reducing morbidity/mortality due to these infections. Another valuable aim is evaluation of pregnancy-induced changes in elephant immune function and response to infectious disease. One report in an Asian elephant dam with a newborn calf suggested that latent *M. tuberculosis* infection was reactivated during late pregnancy or parturition [38]. Altered immune responses during elephant pregnancy may have important implications in the management of these endangered animals.

## 5. Conclusions

Simple laboratory techniques are effective for identification and characterization of Asian elephant monocytes. Phagocytic activity was confirmed and can be quantified via fluorescence microscopy, spectrophotometry, and flow cytometry. The combination of flow cytometric scatter and IBA1 positivity can potentially be used to identify Asian elephant monocytes. These data provide a methodologic basis for further investigation into elephant monocyte function and immune response to infection.

## Figures and Tables

**Table 1:** Primary antibodies and viability dye used in flow cytometry.

Antigen Target	Peripheral WBC Targeted ^1^	Clone	Sequence Similarity ^2^
CD11b	Granulocytes, monocytes	5C6 ^3^	73.5%
CD11c	Monocytes, lymphocytes	3.9 ^4^	72.7%
CD115	Monocytes	12-3A3-1B10 ^4^	79.1%
CD14	Monocytes	Tuk4 ^3^	71.7%
IBA1	Monocytes	1C6A10 ^4^	90.6%
Live/dead	Viability dye (aqua)	N/A ^4^	N/A

IBA1, ionized calcium-binding adapter molecule 1. ^1^ Based on information from other species. ^2^ Sequence similarity listed for each antibody represents comparison of protein sequences between *Loxodonta africana* (African savanna elephant) and human per NCBI BLAST analysis. ^3^ Bio-Rad, Hercules, CA, USA. ^4^ Thermo Fisher Scientific, Waltham, MA, USA.

**Table 2:** Comparison of monocyte and lymphocyte population fractions determined via manual differential counting vs. flow cytometric gating on scatter. Mean values ± standard deviation of three replicates shown. Samples from three elephants (F1, F2, and M1) were assessed.

Elephant	Monocytes (%) via Microscopy	Monocytes (%) via Flow Cytometry	Lymphocytes (%) via Microscopy	Lymphocytes (%) via Flow Cytometry
F1	83.5 ± 2.7	75.3 ± 4.0	16.1 ± 1.9	18.9 ± 2.1
F2	84.0 ± 1.9	76.1 ± 3.3	16.0 ± 1.7	21.2 ± 3.0
M1	82.4 ± 2.3	71.2 ± 2.5	17.5 ± 1.7	22.8 ± 2.5

**Table 3:** Positive events for four surface phenotype markers in monocyte and lymphocyte populations gated via scatter. Mean values ± standard deviation of three replicates shown.

	Positive Events (%)	
	Monocytes	Lymphocytes
CD11b	22.6 ± 3.6	16.7 ± 2.4
CD11c *	47.3 ± 12.8	12.1 ± 3.4
CD115	13.7 ± 1.3	9.4 ± 2.2
CD14	29.5 ± 4.4	21.7 ± 1.5
IBA1 *	86.8 ± 7.5	21.4 ± 3.2

* Significant difference between cell populations, *p* < 0.05.

## Data Availability

The raw data supporting the conclusions of this article will be made available by the authors on request.

## Supplementary Materials

The following supporting information can be downloaded at: https://www.mdpi.com/article/10.3390/ani14162297/s1, Supplementary Methods: Monocyte dissociation buffers; Figure S1: Fluorescence spectrophotometry of elephant monocytes/macrophages exposed to differing concentrations of pHrodo-labeled zymosan particles; Figure S2: Isotype controls for FITC, PE and APC fluorescence used in flow cytometry; Figure S3: Secondary-only controls for FITC and PE used in flow cytometry; Figure S4: Example of gating for IBA1+ events in monocyte populations gated via scatter; Figure S5: Example histogram of overlaid lymphocyte and monocyte populations gated via scatter for CD115-positive events.
