# Supplementary material for: Phagocytic Function and Flow Cytometric Phenotype of Asian Elephant Monocytes
Source: Animals (Basel). 2024 Aug 7;14(16):2297. doi: 10.3390/ani14162297 (PMC11350674; doi:10.3390/ani14162297)

Supplementary Information:

1. Monocyte isolation and dissociation methods: Four dissociation protocols were tested on monocytes isolated in 24-well plates:

   a. Accutase: 0.5 mL of Accutase (Thermo Fisher Scientific) was added per well. Plates were incubated 5, 15, or 30 minutes at 37°C.

   b. Lidocaine buffer: 0.5% bovine serum albumin, 5 mM disodium EDTA, and 4 mg/mL lidocaine were dissolved in DPBS. 0.5 mL of lidocaine buffer was added per well and plates were incubated 15 or 30 minutes at 37°C.

   c. EDTA: 0.5 mM disodium EDTA was dissolved in DPBS. 0.5 mL of EDTA buffer was added per well and plates were incubated 15 or 30 minutes at 37°C.

   d. Dilute trypsin-EDTA: Cell-culture grade 0.25% trypsin with EDTA (Thermo Fisher Scientific) was diluted to 0.05% trypsin in DPBS. 0.5 mL of dilute trypsin-EDTA buffer was added per well and plates were incubated 5, 10 or 15 minutes at 37°C.

Figures:

Suppl. Figure S1: Fluorescence spectrophotometry of elephant monocytes/macrophages exposed to pHrodo-labeled zymosan beads. Values are normalized to “bead only” negative control wells. Monocytes/macrophages from three Asian elephants (female 1 (F1), female 2 (F2), and male 1 (M1) were tested. Data points represent mean values; bars represent standard deviation.

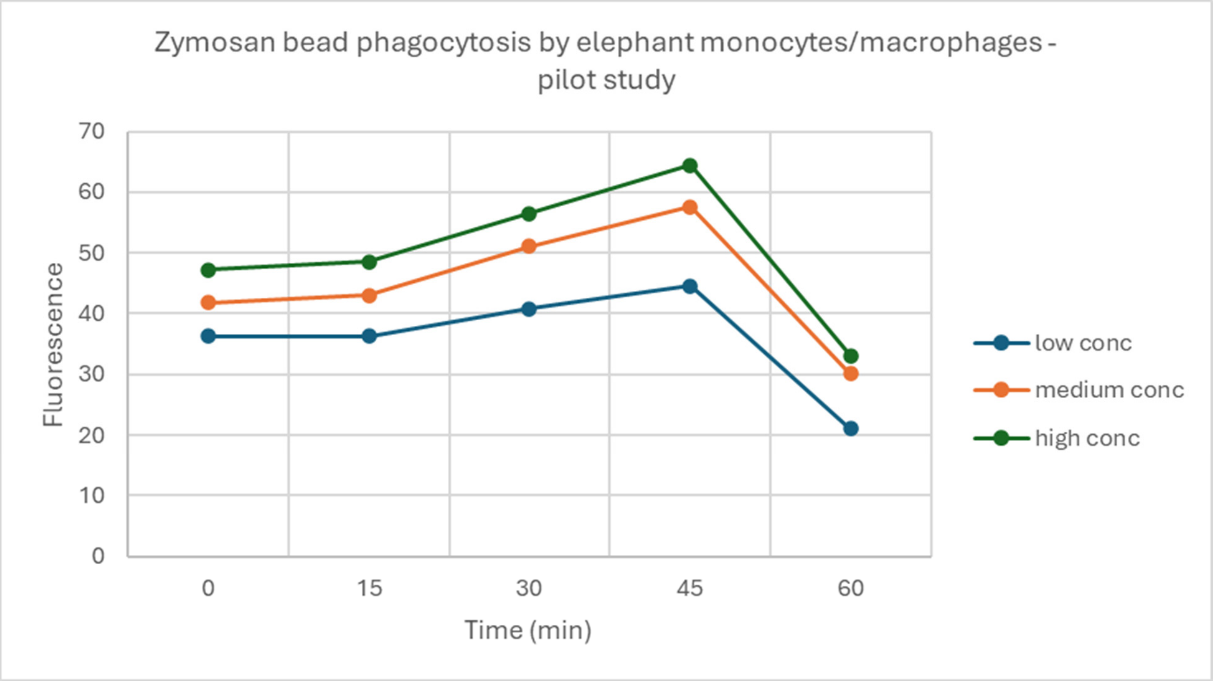

Suppl. Figure S2: Isotype controls for FITC, PE and APC fluorescence used in flow cytometry.

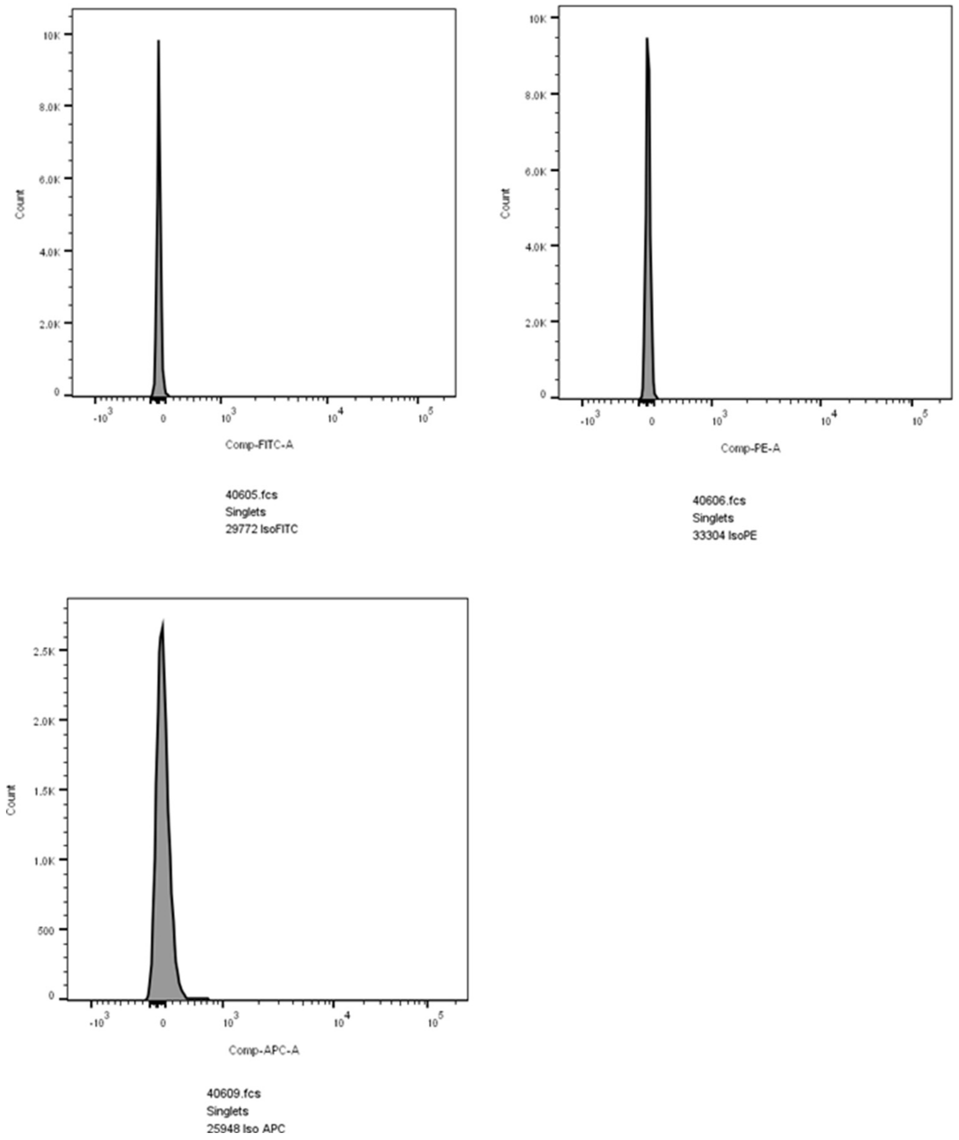

Suppl. Figure S3: Secondary-only controls for FITC and PE used in flow cytometry.

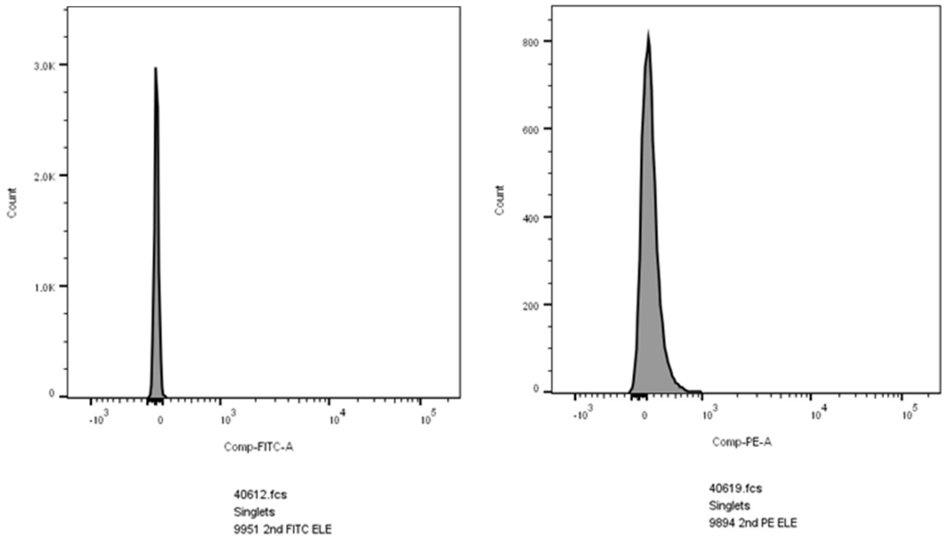

Suppl. Figure S4: Identification strategy for IBA1+ events in monocyte populations gated via scatter. Red peak = FMO (fluorescence minus one) control; blue peak = sample with IBA1 labeling.

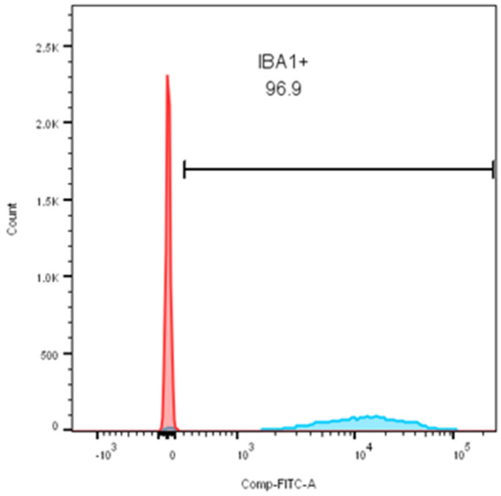

Sample Name	Subset Name	Count
40612.fcs	Monocytes	7870
40632.fcs	Monocytes	7012

Suppl. Figure S5: Example histogram of overlaid lymphocyte and monocyte populations gated via scatter for CD115-positive events. Red peak = lymphocytes; blue peak = monocytes.

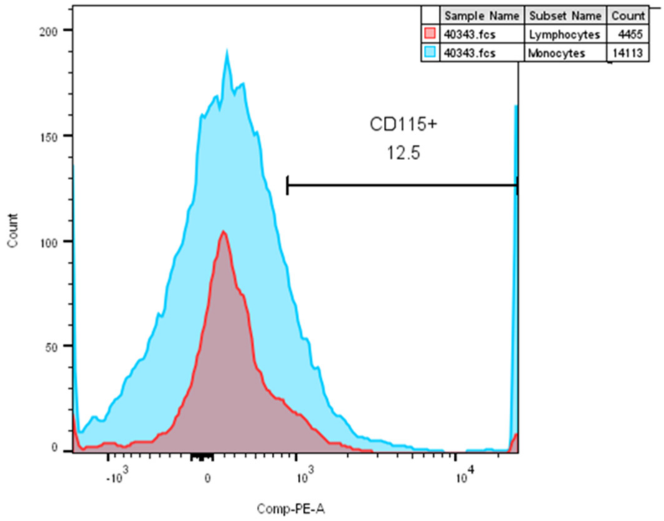

Supplement: Supplementary file 1 [file animals-14-02297-s001.zip › animals-3108666-supplementary.pdf]
